# Slight brightness and osteosclerotic changes of bones on plain X‐ray could be clues to the diagnosis of disseminated carcinomatosis of bone marrow

**DOI:** 10.1002/ccr3.2256

**Published:** 2019-06-11

**Authors:** Masaki Tago, Yoshinori Tokushima, Satsuki Oie, Shu‐ichi Yamashita

**Affiliations:** ^1^ Department of General Medicine Saga University Hospital Saga Japan

**Keywords:** disseminated carcinomatosis of bone marrow, elevated alkaline phosphatase, gastric cancer, plain X‐rays

## Abstract

Clinicians should be alert to the presence of slight brightness and osteosclerotic changes of bones on plain X‐rays, especially in patients without a history of gastric, colon, breast, lung, or prostate cancers, which could lead to the diagnosis of disseminated carcinomatosis of bone marrow.

## 1 CASE

A 34‐year‐old woman with lower back pain was diagnosed with disseminated carcinomatosis of bone marrow due to gastric cancer. Plain X‐rays showed slight brightness and osteosclerotic changes of bones such as disappearance of vertical line of bone trabeculae, which were useful clues for diagnosis.

**Figure 1:**
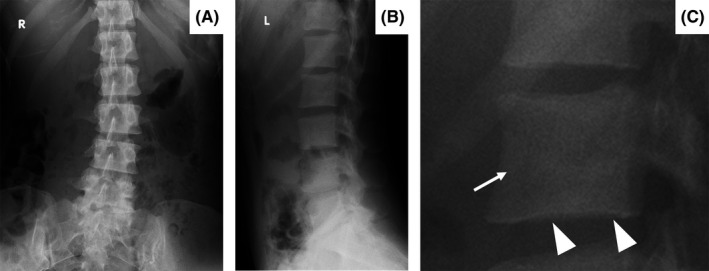
Plain X‐rays of the (A) frontal and (B, C) lateral lumbar spine. Diffuse hyperdense areas are present in the lumbar spine (A, B). The enlarged view of lateral lumbar spines revealed disappearance of vertical lines of bone trabeculae (arrow) and unclear endplates of vertebral body as findings of osteosclerosis (arrowheads)

**Figure 2:**
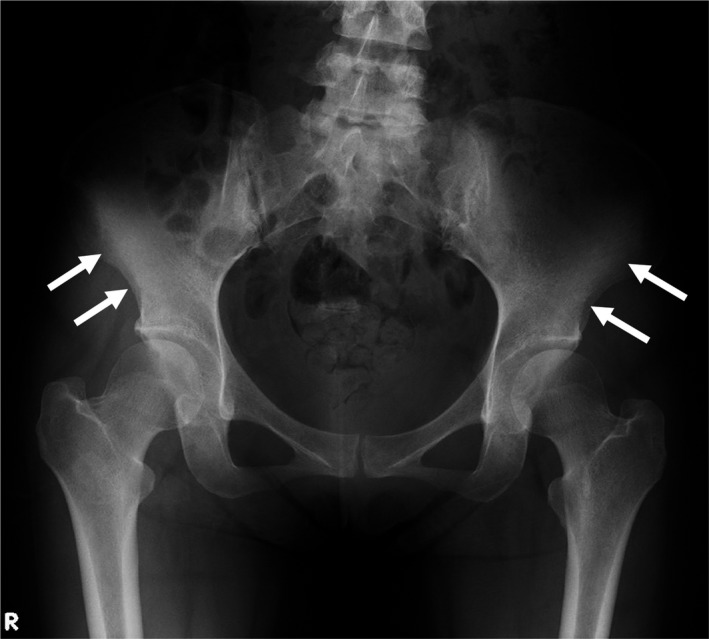
Plain X‐rays of pelvis. Diffuse hyperdense areas are present in bilateral alae of the ilium (arrows)

**Figure 3:**
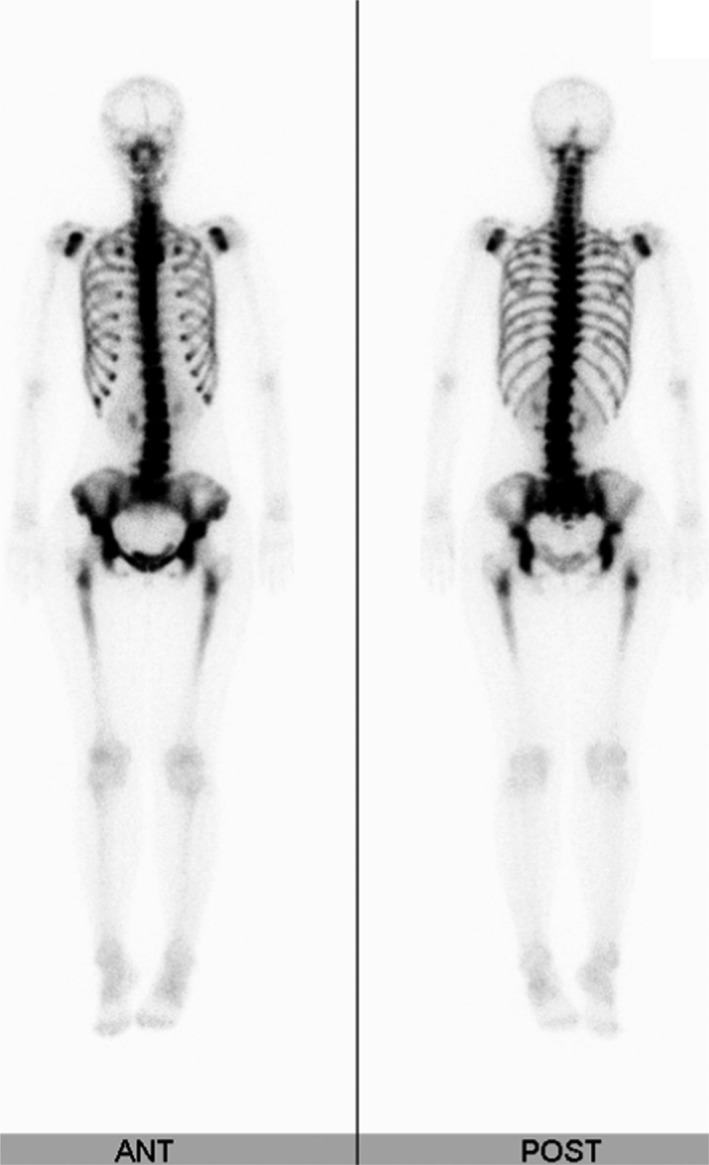
Bone scintigraphy. Bone scintigraphy shows diffuse, even, and dense uptake of radioisotope in spinal and pelvic bones, which is so‐called beautiful bone sign, and absence of both kidneys, absent kidney sign

A 34‐year‐old woman without a history of cancer visited our hospital because of lower back pain. Physical examination showed no abnormalities including breast masses or lymphadenopathy. The laboratory examination showed white blood cell count of 2.9 × 10^9^ cells/L without erythroleukoblastosis, hemoglobin of 9.4 g/dL, platelet count of 136 × 10^9^/L, alkaline phosphatase concentration of 3 196 IU/L (isozyme type 2 plus 3, 91%), and lactate dehydrogenase of 169 U/L. Diffuse hyperdense areas were found in the lumbar spine (Figure 1A,B) and bilateral alae of the ilium (Figure 2). Especially, enlarged view of lateral lumbar spines revealed disappearance of vertical lines of bone trabeculae and unclear endplates of vertebral body as findings of osteosclerosis (Figure 1C). Bone scintigraphy showed beautiful bone sign and absent kidney sign, suggesting diffuse bone metastatic lesions (Figure 3). We suspected disseminated carcinomatosis of the bone marrow (DCBM) caused by gastric or breast cancer; however, upper gastrointestinal endoscopy revealed Borrmann 4 type gastric cancer. We finally diagnosed DCBM due to poorly differentiated gastric adenocarcinoma by histopathological findings of gastric tumor.

Clinicians should be alert to the presence of slight brightness and osteosclerotic changes of bones on plain X‐rays to avoid missing the diagnosis of DCBM, especially in patients without a history of gastric, colon, breast, lung, or prostate cancers.^1^, ^2^

## CONFLICT OF INTEREST

None declared.

## AUTHOR CONTRIBUTION

MT: involved in literature search, study conception, and manuscript drafting. YT: involved in manuscript drafting and clinical care of the patient. SO: involved in literature search and manuscript drafting. SY: involved in study conception and manuscript revision.
